# Paediatric Cogan´s syndrome - review of literature, case report and practical approach to diagnosis and management

**DOI:** 10.1186/s12969-023-00830-x

**Published:** 2023-06-08

**Authors:** Kristina Rücklová, Thekla von Kalle, Assen Koitschev, Katrin Gekeler, Miriam Scheltdorf, Anita Heinkele, Friederike Blankenburg, Ina Kötter, Anton Hospach

**Affiliations:** 1 Klinikum Stuttgart, Kriegsbergstraße 60, 70174 Stuttgart, Germany; 2 Third Faculty of Medicine, Charles University, Prague, Czech Republic; 3 University Hospital Hamburg-Eppendorf, Hamburg, Germany; 4 Clinic for Rheumatology and Immunology Bad Bramstedt, Bad Bramstedt, Germany

**Keywords:** Cogan´s syndrome, Children, Clinical characteristics, Course, Outcome, Practical approach

## Abstract

**Background:**

Cogan´s syndrome is a rare, presumed autoimmune vasculitis of various vessels characterized by interstitial keratitis and vestibular impairment accompanied by sensorineural hearing loss. Due to the rarity of Cogan´s syndrome in children, therapeutic decision making may be challenging. Therefore, a literature search was performed to collect all published paediatric Cogan´s syndrome cases with their clinical characteristics, disease course, treatment modalities used and their outcome. The cohort was supplemented with our own patient.

**Main text:**

Altogether, 55 paediatric Cogan´s syndrome patients aged median 12 years have been reported so far. These were identified in PubMed with the keywords “Cogan´s syndrome” and “children” or “childhood”. All patients suffered from inflammatory ocular and vestibulo-auditory symptoms. In addition, 32/55 (58%) manifested systemic symptoms with musculoskeletal involvement being the most common with a prevalence of 45%, followed by neurological and skin manifestations. Aortitis was detected in 9/55 (16%). Regarding prognosis, remission in ocular symptoms was attained in 69%, whereas only 32% achieved a significant improvement in auditory function. Mortality was 2/55. Our patient was an 8 year old girl who presented with bilateral uveitis and a history of long standing hearing deficit. She also complained of intermittent vertigo, subfebrile temperatures, abdominal pain with diarrhoea, fatigue and recurrent epistaxis. The diagnosis was supported by bilateral labyrinthitis seen on contrast-enhanced magnetic resonance imaging. Treatment with topical and systemic steroids was started immediately. As the effect on auditory function was only transient, infliximab was added early in the disease course. This led to a remission of ocular and systemic symptoms and a normalization of hearing in the right ear. Her left ear remained deaf and the girl is currently evaluated for a unilateral cochlear implantation.

**Conclusions:**

This study presents an analysis of the largest cohort of paediatric Cogan´s syndrome patients. Based on the collected data, the first practical guide to a diagnostic work-up and treatment in children with Cogan´s syndrome is provided.

**Supplementary Information:**

The online version contains supplementary material available at 10.1186/s12969-023-00830-x.

## Background

Cogan´s syndrome (CS) is a vasculitis of various vessels [1] characterized by interstitial keratitis, vestibular impairment resembling Ménière´s disease and progressive sensorineural hearing loss. The interval between the onset of ocular and auditory-vestibular symptoms is usually less than 2 years [2]. Some authors distinguish atypical CS defined by the delay between the ocular and auditory-vestibular manifestations longer than 2 years or with other forms of inflammatory ocular or auditory-vestibular involvement [3].

CS may be accompanied by systemic manifestations, such as fever, weight loss, cardiovascular, musculoskeletal, gastrointestinal or neurological symptoms. The most characteristic cardiovascular complication of CS is an aortitis, which may lead to a significant aortic regurgitation requiring an aortic valve replacement in some cases [4, 5]. The diagnosis of CS is based on clinical criteria and exclusion of alternative causes as summarized in Table 1 (modified according to [6–8].

**Table 1:** Diagnostic criteria and possible differential diagnoses of Cogan syndrome

Mandatory criteria	Clinical manifestations
Auditory-vestibular involvement	Rapidly progressing sensorineural hearing loss
Inflammatory ocular disease	Interstitial keratitis, iridocyclitis, conjunctivitis, episcleritis, anterior and posterior uveitis, retinal vasculitis, acute angle closure glaucoma, papillitis, central vein occlusion, vasculitic optic neuropathy, papilledema
Exclusion of alternative causes	Syphilis, tuberculosis, chlamydia, sarcoidosis, polyarteritis nodosa, ANCA associated vasculitides (granulomatosis with polyangiitis, eosinophilic granulomatosis with polyangiitis and microscopic polyangiitis), Behcet´s disease, Takayasu arteritis, Vogt-Koyanagi-Harada syndrome, Susac syndrome, connective tissue disorders
Possible additional criteria	
Nonspecific systemic symptoms	Fever, weight loss, fatigue, lymphadenopathy
Neurological	Headache, hemiparesis or hemiplegia, aphasia, Encephalitis, peripheral neuropathy
Vestibular	Vertigo, dizziness, tinnitus, ataxia
Cardiovascular	Aortitis, vasculitis of other vessels including coronary, renal or mesenteric arteries, peri/myocarditis
Musculoskeletal	Arthralgias/arthritis, myalgias/myositis
Gastrointestinal	Diarrhoea, rectal bleeding or melena, abdominal pain, peptic or colonic ulcerations, hepatosplenomegaly
Laboratory	Increased systemic inflammatory markers

So far, only approximately 300 adults with CS have been reported (Orphanet) and it is even more rare in children. Hence, counselling as well as therapeutic decision making may be challenging especially in the paediatric population. The aim of this study is to provide a comprehensive overview of the course and outcome of CS in childhood and to offer a practical guide for initial diagnostic work-up and treatment based on the largest collection of paediatric cases from literature so far. The cohort is supplemented by our own patient that is rather unique due to her low age and a successful treatment with infliximab.

## Main text

### Case report

A previously healthy 8-year-old girl of non-consanguineous Rumanian origin was referred to our department with a 4-week history of anterior uveitis. This manifested as redness, decreased visual acuity and photophobia and initially responded well to topical steroids. In addition, hearing difficulties could be retrospectively discerned for several preceding months. A severe (left ear) and a moderate (right ear) sensorineural hearing deficit was subsequently confirmed by an audiometry and a brain stem evoked response audiometry (BERA). During the course of the disease the patient developed intermittent vertigo, low-grade fever, recurrent headache, abdominal pain, diarrhoea with rectal bleeding and repeated epistaxis.

**Fig. 1:**
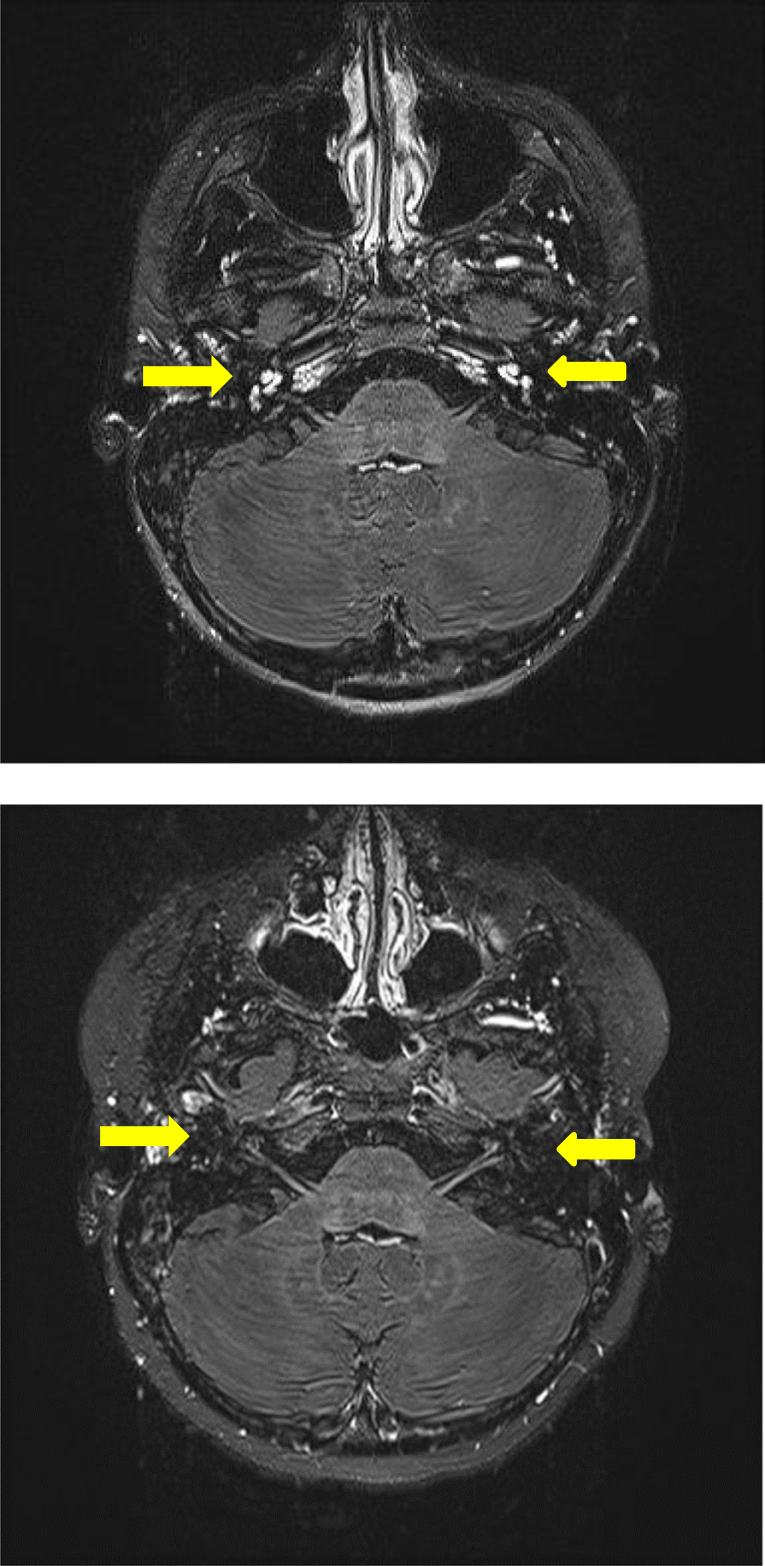
MR images in STIR sequences showing a) florid labyrinthitis before treatment and b) absence of labyrinthitis after 4 months of immunomodulatory treatment

Laboratory investigations revealed low inflammatory markers (CRP < 1 mg/l, ESR 17 mm/h), normal CBC (WBC 7,730/µl, Hb 12 g/dl and PLT 435,000/µl), normal electrolytes, creatine, urinalysis and liver enzymes. Workup for infections, including screening for CMV, Chlamydia, Syphilis, Lyme disease, Mycoplasma and Mycobacterium tuberculosis, was negative. The following tests were also negative or normal: ANA, ANCA, RF, LA, β2-GP and aCL antibodies, serum angiotensin-converting enzyme and calprotectin in stool. A chest X-ray, MRI of the brain and cerebrospinal fluid were normal as well. A whole-exome sequencing (WES) was indicated to exclude inborn disorders of hearing and revealed no relevant pathogenic variants. The combination of uveitis and sensorineural hearing deficit led to a diagnosis of CS. In addition, a contrast-enhanced MRI of the inner ear was performed and showed signal enhancement in the labyrinth and cochlea bilaterally raising suspicion of a bilateral labyrinthitis, a finding compatible with CS Fig. 1. An echocardiography excluded aortic dilatation.

**Fig. 2:**
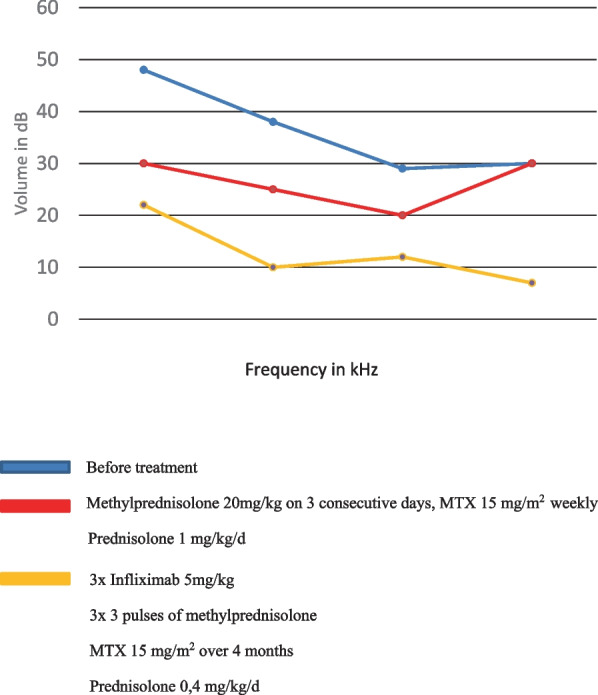
Pure tone audiogram of the right ear under various treatment modalities

Therapy was started with i.v. methylprednisolone (20 mg/kg, 3 doses over 3 consecutive days) repeated four times at 5-week intervals, followed each time by oral prednisolone at 1 mg/kg/day, methotrexate at 15 mg/m^2^ weekly and topical steroid eyedrops. The uveitis regressed within 4 weeks. However, the patient developed a pronounced bilateral keratitis and had to be restarted on topical steroids and ciclosporin. As for her hearing capacity, she initially responded with a transient improvement in hearing. During further course of the disease the auditory function deteriorated again. Within 2 months the patient developed a secondary glaucoma and Cushing signs necessitating a reduction of the systemic steroid treatment. Additionally, infliximab (5 mg/kg at 2-week intervals initially, currently at 4-week intervals) was started with the aim to restore hearing. The auditory function normalized in the right ear as confirmed on the pure tone audiogram (Fig. 2), whereas the left ear remained deaf during the 8-month follow-up and the patient is being evaluated for unilateral cochlear implantation. During follow-up we could also confirm resolution of the inflammation within labyrinth and cochlea bilaterally (Fig. 1). This fact may, however, indicate intracochlear fibrosis on the deaf side and emphasize the urgency of CI. The inflammatory eye involvement resolved completely leaving posterior synechia. The patient still suffers from intermittent balance disorder, fatigue and recurrent epistaxis.

### Review of literature

In order to provide the family of our patient with an evidence-based counselling and to support our therapeutic decision making, we performed a review of literature and identified altogether 55 paediatric CS case reports in PubMed under the keywords: “Cogan syndrome” and “children” or “childhood”. The clinical characteristics, treatment and outcome are presented in Table 2 and Supplementary material 1 [2–4, 8–42]. All the reported patients had sensorineural hearing deficit and ocular involvement with interstitial keratitis being the most common form detected in 62%, followed by uveitis found in 36%. Papilledema was seen in 3/55 patients. Vestibular symptoms included vertigo or tinnitus sometimes associated with vomiting and were reported in 69% of patients. Systemic symptoms such as fever, arthralgias/arthritis, neurological or skin involvement were reported in 29, 45, 31 and 18%, respectively. Among neurological symptoms, headache was the most commonly described complaint, however, meningitis and facial nerve palsy have also been reported in single cases [4, 8, 40]. Skin lesions included nodular, urticaria-like lesions, furuncles and ulceration at the site of TBC vaccination [10, 17, 19, 29–32, 35, 37, 40]. Rarely, lymphadenopathy or hepatosplenomegaly, weight loss or anorexia have been reported [2, 8, 10, 12, 22, 27, 28, 31]. Renal involvement classified as glomerulonephritis or tubulointerstitial nephritis have been reported in three children [4, 8, 26]. The most common potentially fatal cardiovascular complications included an aortitis identified in 9/55 (16%), three of them required a surgical intervention, and a pericarditis found in 2/55 (4%) patients [2, 4, 10, 26, 27, 36–39]. Two patients out of the whole cohort died. One was a 14 year old boy reported by Cogan in 1964. The patient received no systemic treatment and succumbed to an aortitis with a massive mural thrombus, aortic insufficiency and heart failure [2]. The second patient suffered from steroid dependent CS and died from a subarachnoid haemorrhage despite of treatment with high dose systemic steroids and cyclophosphamide [30].

**Table 2:** Clinical characteristics, treatment and treatment outcomes of paediatric CS patients. Patients from the literature and our patient. N = 55

Number of patients	55
Age, years, median (min–max)	12 (3–18)
Gender, male, n (%)	Male 31/54 (57)
Follow-up period, years (min–max)	2 (0.2–16)
Clinical findings, n (%)	
Ocular findings	55/55 (100)
Interstitial keratitis	34/55 (62)
Uveitis	20/55 (36)
Conjunctivitis	10/55 (18)
Sensorineural hearing deficit	55/55 (100)
Vestibular symptoms	37/54 (69)
Any systemic symptoms	32/55 (58)
Fever	16/55 (29)
Musculoskeletal symptoms	25/55 (45)
Neurological signs	17/55 (31)
Aortitis	9/55 (16)
Skin involvement	10/55(18)
Gastrointestinal symptoms	6/55 (11)
Elevated CRP and/or ESR	29/36 (81)
Treatment, n (%)	
Systemic corticosteroids	44/54 (81)
MTX	14/54 (26)
AZA	5/54 (9)
CYC	5/54 (9)
MMF	3/54 (6)
Cyclosporine	3/54 (6)
Cochlear implantation	7/55 (13)
Outcome, hearing, n (%)	
Deafness/ unilateral deafness/ severely impaired	28/50 (56)
Moderately impaired	6/50 (12)
Complete/partial remission/mildly impaired	16/50 (32)
Outcome, ocular symptoms, n (%)	
Blindness/ unilateral blindness	2/48 (4)
Decreased visual acuity	5/48 (10)
Recurrences	6/48 (13)
Remission	33/48 (69)
Mortality	2/55 (4)

The most common treatment by far were systemic corticosteroids used in 81%, followed by methotrexate in 26% of patients. Only 2 paediatric patients including ours have been treated with biologics so far, both of them with infliximab [19]. The outcome of ocular involvement has been shown much more favourable in comparison to the auditory-vestibular function with 68% of patients remaining deaf, severely or moderately affected.

## Discussion

So far no diagnostic and management recommendations for CS in children have been published. Unfortunately, there is no single laboratory test that would be diagnostic for CS. Although autoantibodies against an autoantigen DEP-1/CD148 have been identified in patients with CS, they are not routinely available. The autoantigen DEP-1/CD148 is present on endothelium, nerve and glial cells as well as within inner ear thereby explaining the multiple organ damage in CS patients. In addition, these antibodies cross-react with a structural protein of the Reovirus type III, suggesting that this or similar infections may be involved as trigger in the pathogenesis of CS [43]. Apart from DEP-1/CD148 autoantibodies, anti-heat shock protein 70 antibodies have been detected in a substantial proportion of patients with CS [44].

As DEP-1/CD148 and anti-heat shock protein 70 autoantibodies are not routinely tested, the diagnosis of CS has to rely on a clinical suspicion and a constellation of results from various diagnostic methods. Most of them are meant to exclude another alternative diagnosis. Based on our experience and the reported cases we provide a rational diagnostic work-up for children with suspected CS (Table 3). Firstly, inflammatory markers should be included in the initial diagnostic work-up as they were increased in more than 80% of the reported paediatric patients. Electrolytes, renal and hepatic parameters together with urinalysis should be included to assess possible organ involvement. We also suggest performing a basic autoimmune work-up with ANA, ANCA, ENA, RF, complement and antiphospholipid antibodies to help differentiate other rheumatological conditions such as e.g. systemic lupus erythematosus and ANCA associated vasculitides. On the other hand, positivity in at least one of these parameters has been detected in 10 children with CS and no alternative diagnosis. The positivity may therefore be regarded as rather non-specific [12–16, 26–28]. Cerebrospinal fluid pleocytosis has been reported in 5/14 patients who had undergone lumbar puncture [4, 21, 22, 33, 40]. Three of them had documented neurological complaints. In our opinion cerebrospinal fluid analysis may be reasonable to document central nervous system involvement and it should definitely be performed in all cases where neuroinfection is clinically suspected.

**Table 3:** Suggested initial diagnostic work-up for paediatric patients with suspected Cogan's syndrome

Investigation	Comment
Laboratory: Inflammatory markers CBC Electrolytes, creatinine, liver tests, urinalysis Infectious work-up ACE, s-IL2R, Ca in urine T-spot ANA, ENA, ANCA C3, C4, CH50	Mostly elevated Often anaemia, eosinophilia, increased WBC and PLT To detect possible multiorgan involvement or alternative diagnosis To exclude Chlamydia trachomatis, Treponema pallidum, Lyme disease, toxoplasmosis, CMV, EBV, Mycoplasma To exclude sarcoidosis To exclude tuberculosis To exclude systemic lupus erythematodes, ANCA- associated vasculitis
Audiometry	To diagnose and monitor hearing deficit
BERA	To confirm sensorineural hearing deficit especially in children unable to cooperate during audiometry
Vestibular evoked myogenic potentials Video head impulse testing Rotary chair testing Caloric testing	To asses vestibular function
Ophthalmologic investigation including slit lamp	To detect interstitial keratitis, uveitis, papilledema, synechia as long-term consequences of uveitis, secondary glaucoma as a result of corticosteroid therapy and to monitor visual acuity
MRI brain and inner ear with gadolinium	To exclude cerebellopontine tumours or stroke Enhancement of cochlea and labyrinth with gadolinium as signs of inflammation may support the diagnosis Pre-operative assessment before CI (may need to be supplemented by HRCT)
Echocardiography	To detect and monitor aortic root dilatation and aortic regurgitation
Cerebrospinal fluid in case of neurological symptoms	To exclude meningoencephalitis of infectious or other etiology

Regarding imaging, an echocardiography must be indicated in all patients with suspected CS as an aortitis and a pericarditis represent the most common potentially lethal complications. A follow-up echocardiography may be reasonable once yearly in patients in remission and more frequently in patients with active inflammation. Furthermore, a contrast-enhanced MRI of the brain and the inner ear should be recommended. Labyrinthitis has been shown in 5 children with CS [17–19, 22]. Its presence may support the inflammatory nature of the auditory-vestibular involvement while excluding neoplasm or ischaemia*.*

As far as treatment is concerned, there is a consensus from adult cohorts that mild ocular involvement may be treated by topical steroids, whereas severe eye inflammation, inner ear disease and systemic manifestations require systemic immunosuppressive agents with steroids representing the mainstay of treatment [45, 46]. The beneficial effect of treatment has been documented mainly for ocular and vestibular involvement, whereas severe hearing deficit persists in approximately half of adult patients [5]. Reports on the efficacy of conventional disease-modifying anti-rheumatic drugs (cDMARDs) and biologics are mostly anecdotal except for one larger scale study [47] on 62 patients. From these, 10 were treated with infliximab, which was shown to be significantly superior to other drugs in improving the auditory function.

In agreement with the data from adults, in the presented paediatric cohort the auditory response to therapy was rather poor with only 32% patients achieving complete or at least partial remission. Clinical outcome with respect to different treatment modalities is demonstrated in Table 4. Regarding ocular involvement, remission has been achieved in 50–79% patients irrespective of treatment modality, even though the use of systemic CS seem to increase the response rate to 79%. As far as hearing is concerned, the absence of systemic treatment has been shown significantly inferior to any systemic treatment. None of the patients in the group without systemic therapy attained remission in auditory function. On the other hand, complete or partial remission in hearing has been shown in 57, 27 and 50% of paediatric patients treated with systemic CS only, systemic CS plus cDMARDs and systemic CS, cDMARDs plus biologics, respectively. The small number of patients in our study does not allow for statistical comparison among the subgroups.

**Table 4:** Clinical outcome with respect to systemic treatment modality. Patients from the literature supplemented by our patient. *N* = 55

Treatment modality	Outcome		
	Auditory	Ocular	Other
No of patients (%)			
No systemic treatment *N* = 11/55 (20)	Deaf: 7/11 (64) Moderately impaired: 3/11 (27) Remission: 0/11 (0)	Recurrences 2/11 (18) Remission 6/11 (55)	Death 1/11 (9) Arthritis 1/11 (9)
Systemic CS *N* = 14/55 (25)	Deaf/severely impaired: 6/14 (43) Complete/partial remission: 8/14 (57)	Impaired vision 2/14 (14) Remission 11/14 (79)	Arthritis 1/14 (7)
Systemic CS and cDMARDs *N* = 26/55 (47)	Deaf/severely impaired 11/26 (42) Moderately impaired: 7/26 (27) Complete/partial remission: 7/26 (27)	Impaired vision: 3/26 (12) Recurrences: 4/26 (15) Secondary complications: 2/26 (8) Remission: 14/26 (54)	Death 1/26 (4) Vestibular 2/26 (8) Vascular 1/26 (4) Renal 2/26 (8)
Systemic CS, cDMARD and biologics *N* = 2/55 (4)	Severely impaired 1/2 (50) Complete/partial remission: ½ (50)	Decreased vision: 1/2 (50) Remission: ½ (50)	None
Systemic CS and colchicine *N* = 2/55 (4)	Deaf/severely impaired: 2/2 (100)	Decreased vision: ½ (50) Remission: ½ (50)	Vestibular: ½ (50)

Conventional DMARDs included: MTX, AZA, CsA, MMF, CYC. Secondary ocular complications included a glaucoma and a cataract. All patients received topical steroids

Importantly, auditory improvement with pharmacological treatment may be expected only in early stages of the disease before the development of irreversible fibrosis and osteoneogenesis within the organ of Corti [48]. For patients, whose hearing could not be salvaged by pharmacological methods, cochlear implantation (CI) offers an excellent option [49].

Within the adult CS population, 51 patients have been implanted. Almost half of these patients (23/50, 46%) had a cochlear ossification, which may pose a surgical challenge. However, this obstacle could be managed in all patients by tunnelling through the ossified portion or scala vestibuli electrode insertion. Only 4/51 (8%) patients experienced complications related to the procedure and 6/51 (12%) developed a relapse of CS (interstitial keratitis in 4 and systemic symptoms in 1) immediately after the surgery. Excellent word and sentence recognition scores were reported in 43/46 (93%) after CI [50–64]. In the present cohort of paediatric CS patients, CI has been shown feasible and beneficial. Altogether 7 paediatric CS patients have undergone a successful CI without complications [9, 10, 19, 42].

Another reported therapeutic modality for sudden hearing loss are intratympanic steroid injections. These allow for achievement of higher concentrations of steroids in the cochlear fluid avoiding negative side effects of high dose systemic therapy [65]. However, the procedure is associated with a risk of inducing acute middle ear infections or persistent perforation of the tympanic membrane. In addition, there is little evidence for its long-term efficacy in CS patients. In a study done by Parnes et al. 2 patients with CS have undergone repeated intratympanic steroid injections. A clear long-term benefit has been demonstrated in one of them, whereas the other one showed only a transient improvement. The latter developed repeated middle ear infections as a result of the procedure [65]. Looking at our paediatric data, intratympanic steroid injections have been reported in a single child with CS and resulted in no benefit [19].

Given the above mentioned data from adult and our paediatric cohorts, we decided for an aggressive anti-inflammatory treatment in our patient. This included high-dose steroids and methotrexate, which led to an impressive but short-lasting improvement of hearing.As she developed pronounced Cushing signs and a secondary glaucoma, steroids had to be reduced while stepping up the treatment with another agent. Although infliximab has only been used once in a paediatric CS patient [19] with no benefit for hearing, based on the robustness of the data from the above-mentioned adult study [47] we decided for an early addition of infliximab. This had an excellent effect on the ocular involvement and the hearing in the right ear (Fig. 2). The left ear remained deaf presumably due to a long period between the onset of hearing deficit and treatment and CI is currently being considered.

## Conclusions

To conclude, the outcome of paediatric CS patients proved good regarding their ocular involvement with 69% achieving remission but rather unsatisfactory with respect to hearing deficit with 56% children remaining deaf or severely affected. These findings may underline the need to start early and aggressive systemic treatment in children with an auditory deficit. Systemic steroid and infliximab treatment seem to be the most evidence-based pharmacological interventions although the data supporting the use of infliximab are derived solely from a study on adults. In cases of persistent severe hearing loss, CI should be performed as it is considered a safe and efficient option even in children. It has been shown feasible even in the terrain of ossified cochlea. Data supporting the use of intratympanic steroid injections in children with CS are minimal.

Our case supplements the current knowledge on paediatric CS and is unique due to the relatively young age of our patient (8 vs. median 12 years), absence of increased inflammatory markers (observed in 81% of paediatric CS patients) and successful use of infliximab. Based on the review of literature we suggest a rational diagnostic work-up for paediatric CS that should include laboratory autoimmune and infectious screening as well as echocardiography and MRI of the brain and the inner ear with gadolinium enhancement.

## Supplementary Information

**Additional file 1.**

## Data Availability

All the data have been stored within the archive of our hospital and may be presented upon request.

## Abbreviations

ANA: Antinuclear antibodies
ANCA: Anti-neutrophil cytoplasmic antibodies
AZA: Azathioprine
BERA: Brainstem evoked response audiometry
β2-GP: β2-Glycoprotein
CBC: Complete blood count
sDMARD: Conventional disease modifying anti-rheumatic drug
CI: Cochlear implantation
CL: Cardiolipin
CMV: Cytomegalovirus
CRP: C-reactive protein
CS: Cogan syndrome
CsA: Ciclosporin A
CYC: Cyclophosphamide
DMARD: Disease modifying anti-rheumatic drug
ENA: Extractable nuclear antigens
ESR: Erythrocyte sedimentation rate
Hb: Haemoglobin
LA: Lupus anticoagulans
MMF: Mycophenolate mofetil
MRI: Magnetic resonance imaging
MTX: Methotrexate
PLT: Platelets
RF: Rheumatoid factor
TBC: Tuberculosis
WBC: White blood cells
WES: Whole-exome sequencing
